# Management of skin metastases of ovarian cancer: a novel therapeutic approach

**DOI:** 10.1007/s00066-026-02546-9

**Published:** 2026-05-13

**Authors:** Anne-Marie Lüchtenborg, Jörg Sahlmann, Kristin Technau-Hafsi, Natalia Volegova-Neher, Beate Rautenberg, Peter Vaupel, Anca-L. Grosu, Andreas R. Thomsen

**Affiliations:** 1 Department of Radiation Oncology, Medical Center – University of Freiburg, Faculty of Medicine, University of Freiburg, Robert-Koch-Strasse 3, 79106 Freiburg, Germany; 2 Institute for Medical Biometry and Statistics, Medical Center – University of Freiburg, Faculty of Medicine, University of Freiburg, Freiburg, Germany; 3 Department of Dermatology, Medical Center – University of Freiburg, Faculty of Medicine, University of Freiburg, Freiburg, Germany; 4 Department of Obstetrics and Gynecology, Medical Center – University of Freiburg, Faculty of Medicine, University of Freiburg, Freiburg, Germany

**Keywords:** Hyperthermia, Combined modality therapy, Cutaneous metastases, Hypofractionated radiotherapy, Palliative care, Water-filtered infrared A (wIRA)

## Abstract

**Purpose:**

Ovarian cancer, one of the most lethal gynecological cancers, metastasizes into skin in 0.9%–5.8% of cases. Cutaneous metastases severely affect the quality of life of ovarian cancer patients. Although cutaneous metastases are rare, a therapeutic option for affected patients is needed. Herein, we present a combination therapy comprising radiation and mild hyperthermia with parallel chemotherapy as a treatment modality.

**Case:**

A woman with extensive skin metastases of a high-grade, serous ovarian carcinoma on the thigh was treated with a combination of mild hyperthermia (39–43 °C) immediately followed by low-dose hypofractionated radiotherapy and parallel systemic treatment with carboplatin/gemcitabine. Mild hyperthermia, a strong radiosensitizer, was induced through water-filtered infrared A radiation (wIRA).

**Results:**

The patient responded well and remained tumor free in the treatment area for more than 1 year.

**Conclusion:**

Radiotherapy combined with mild hyperthermia in addition to systemic treatment allows for tumor control in the treated area, even with a reduced total radiation dose. To the best of our knowledge, this is the first report of a long-term tumor-free situation in the treatment area of skin metastases of ovarian cancer. This novel treatment might also be beneficial for skin metastases from other malignancies.

## Introduction

Ovarian cancer accounted for 314,000 new cases and > 207,000 deaths worldwide in 2020 and remains one of the most lethal gynecological cancers due to late diagnosis and a high recurrence rate [1]. Metastasis occurs through direct dissemination into the peritoneum as well as via hematogeneous, lymphatic, and neural pathways [2]. Cutaneous metastases of ovarian cancer are rare (0.9%–5.8% of cases), typically manifesting in the abdomen and limbs, likely due to lymphatic spread [3, 4]. They appear late in the disease course but represent a significant burden on quality of life. In the palliative setting, successful treatment of extensive cutaneous metastases is critical [4], and satisfying options are missing so far, often due to previous extensive therapy or the extent of metastasis.

## Case

This case describes a woman diagnosed with a high-grade serous ovarian carcinoma at the age of 39 years (FIGOIIIC, pT2a, pN1 [10/12], L1, V1, Pn0, G3, *BRCA1* + *BRCA2*: negative). Initial treatment comprised extensive but incomplete resection (R2) and chemotherapy with carboplatin/paclitaxel and bevacizumab. Thirty-two months after the initial diagnosis, the patient developed a first progression, followed by multiple metastases (lymph nodes, cerebral) over the next few years; these were treated with systemic therapy, excision, and radiation therapy, resulting in stable disease for 9 years.

**Fig. 1:**
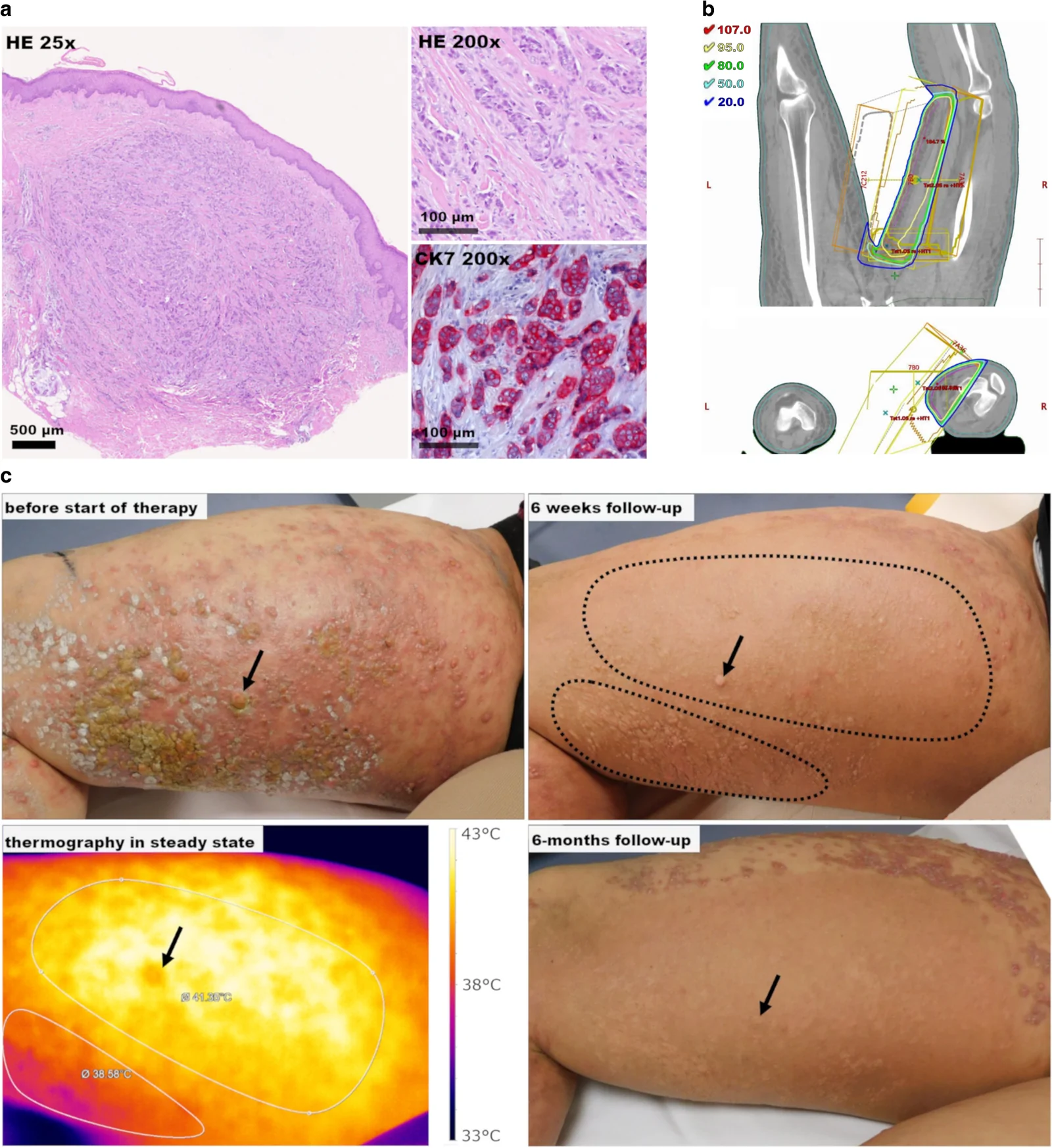
**a** Histology of skin biopsy: Hematoxylin and eosin (*H&E*; left × 25, top right × 200) and CK7 (bottom right × 200) immunohistochemistry staining confirming metastases with signs of lymphatic vessel invasion. **b** Radiation plan with isodoses (% of prescribed dose) demonstrates the large treatment field. **c** Clinical outcome: initial diagnosis of extensive skin metastases from ovarian cancer (top left), strong regression at the 6‑week follow-up (top right), and complete disappearance of evidence of disease at the 6‑month follow-up (bottom right). Representative thermography image in thermal steady state of hyperthermia treatment (bottom left) demonstrated an uneven temperature distribution within the anterior and posterior subregions (outlines). Arrow represents a prominent nodule for orientation

Nine and a half years after the initial diagnosis, the patient presented with a progressive, extensive, multi-locular rash on the thoracic region, mons pubis, and both thighs during chemotherapy with carboplatin/gemcitabine and reported needle prick-like pain and itching in the affected areas. The lesions were first suspected to be herpes zoster but did not respond to acyclovir. Biopsy confirmed skin metastases of the ovarian cancer, with signs of lymph invasion (Fig. 1a). Due to previous pelvic and inguinal irradiation, conventionally fractionated re-irradiation was not indicated. Analogous to the established protocol for nonresectable breast cancer recurrences [5], the patient was offered hypofractionated radiotherapy combined with mild hyperthermia (mHT + RT) as a local treatment option in parallel to the ongoing systemic therapy with carboplatin/gemcitabine. Mild, superficial hyperthermia was induced with water-filtered infrared A (wIRA) irradiation under thermographic temperature control (TWH 1500, Hydrosun™, Müllheim, Germany) for 60 min immediately before radiation, allowing for clinically relevant hyperthermia levels up to 2.5 cm depth [6, 7].

Linac-based radiation therapy with 4 Gy once weekly (total dose 20 Gy) was delivered by tangentially opposed photon fields (6 MV) that were three dimensionally planned to the part of the right thigh that was most severely affected by cutaneous metastases (Fig. 1b). A pre-warmed 5‑mm silicone bolus was applied to the thigh, and the patient position was adjusted via cone-beam CT.

Superficial tissue heating to a maximum of 43 °C was controlled via thermography to avoid tissue damage. Given the geometry of the thigh and the large area to be heated, the treatment field exhibited an inhomogeneous temperature distribution, with significantly higher temperatures in the anterior versus the posterior sub-region (Fig. 1c). Over all treatments, the mean steady-state temperature was 41.2 °C in the ventral sub-region (T90, 40.7 °C; T10, 41.8 °C) and 39.2 °C in in the medial/dorsal part (T90, 38.0 °C; T10, 40.4 °C). The thermal dose, calculated as Cumulative Equivalent Minutes at 43°C (CEM43), delivered to the posterior sub-region was only 5%–7% of the thermal dose in the anterior sub-region across all five treatment sessions.

The patient responded very well to mHT + RT (Fig. 1c). After a dose of 12 Gy, the ulcerating efflorescence diminished, and the skin recovered. Interestingly, metastases in the well-heated anterior sub-region regressed earlier than those in the posterior sub-region. Six weeks after the end of treatment, the metastases continued to recede; a clinical complete response—defined as macroscopic disappearance of the lesion on physical examination—was observed in the dorsal area with complete resolution of associated symptoms, and a strong regression in the colder, posterior area (Fig. 1c). After 6 months, the metastases had disappeared completely across the treated field. In the non-treated area, skin metastases continued to spread; the patient was offered additional treatment fields on the upper and lower leg, thoracic wall, and mons pubis, with similar success. The treated area remained metastases free until death due to systemic progression 13 months after completion of the first mHT + RT treatment.

## Discussion

To our knowledge, this is the first report of successful treatment of extensive cutaneous metastases of ovarian cancer that provided long-term, metastasis-free survival in the treatment area and a good palliative outcome.

To date, only few cases of patients with highly extensive cutaneous metastases from ovarian cancer have been published [4, 8–12]. Treatment options were chemo- and/or radiotherapy, as surgery was not indicated, mainly due to the large sizes of the metastases. In all reported cases, only limited responses or a relapse after initial improvement were observed. One reported case was treated with carboplatin/gemcitabine and 24-Gy radiotherapy without HT, similar to the patient described here. The patient experienced minimal therapeutic benefit, underscoring the radiosensitizing effect of mHT treatment [4].

Mild hyperthermia is usually applied in a temperature range of 39–43 °C. Using thermographic control of the skin, two areas with a temperature difference of 2 °C (39.2 vs*.* 41.2 °C) were defined. In several clinical studies with HT + RT or HT + chemotherapy, higher tissue temperatures resulted in better outcomes [13]. Here, higher temperatures seemed to translate into a faster clinical response. Notably, adjacent areas exposed to elevated temperatures from wIRA alone, without concurrent RT, showed no response, highlighting the necessity of the combination therapy.

Although chemotherapy was administered concurrently over the course of mHT + RT, carboplatin/gemcitabine was not given simultaneously with or at short intervals before mHT + RT to reduce toxicity. This limits the synergistic effects between carboplatin/gemcitabine and mRT + HT due to drug elimination. However, in the third chemotherapy cycle, HT was administered 24 h before carboplatin/gemcitabine. This interval may potentiate the drugs’ cytotoxic effects by HT-induced transient suppression of DNA repair mechanisms such as homologous recombination, thus increasing tumor cell sensitivity to chemotherapy [14, 15]. It is apparent that chemotherapy alone was not sufficient to control disease, as metastatic progression occurred in regions that did not receive mHT+RT.

Skin metastases usually present at advanced disease stage and are associated with a poor prognosis. In a small series of skin metastases from ovarian cancers, Cormio et al. reported a median survival of 4 months after the diagnosis of skin metastases and surgical resection and/or chemotherapy with lesions up to 2 cm in diameter [16].

In conclusion, mHT immediately followed by RT is a suitable treatment option for patients with extensive skin metastases from ovarian cancer and might be similarly beneficial for other superficial cancer entities (e.g., melanoma, angiosarcoma, basal cell carcinoma).

## Data Availability

All relevant data are included within the article. Additional clinical data are available from the corresponding author on reasonable request, subject to patient confidentiality.
